# Combined DNA, toxicological and heavy metal analyses provides an auditing toolkit to improve pharmacovigilance of traditional Chinese medicine (TCM)

**DOI:** 10.1038/srep17475

**Published:** 2015-12-10

**Authors:** Megan L. Coghlan, Garth Maker, Elly Crighton, James Haile, Dáithí C. Murray, Nicole E. White, Roger W. Byard, Matthew I. Bellgard, Ian Mullaney, Robert Trengove, Richard J. N. Allcock, Christine Nash, Claire Hoban, Kevin Jarrett, Ross Edwards, Ian F. Musgrave, Michael Bunce

**Affiliations:** 1 Trace and Environmental DNA laboratory, Department of Environment and Agriculture, Curtin University, Kent St, Bentley, WA, 6102, Australia; 2 Separation Science and Metabolomics Laboratory and the Advanced Mass Spectrometry Facility, Murdoch University, South St, Murdoch, WA, 6150, Australia; 3 School of Veterinary and Life Sciences, Murdoch University, South St, Murdoch, WA, 6150, Australia; 4 School of Medical Sciences, The University of Adelaide, Frome Rd, Adelaide, SA, 5005, Australia; 5 Forensic Science SA, Adelaide, SA, 5000, Australia; 6 Centre for Comparative Genomics, Murdoch University, South St, Murdoch, WA, 6150, Australia; 7 LotteryWest State Biomedical Facility Genomics, School of Pathology and Laboratory Medicine, University of Western Australia, 35 Stirling Hwy, Crawley, WA, 6009, Australia; 8 Department of Diagnostic Genomics, Pathwest Laboratory Medicine WA, QEII Medical Centre, Hospital Ave, Nedlands, WA, 6009, Australia; 9 Trace Research Advanced Clean Environment (TRACE) Facility, Department of Physics, Astronomy and Medical Radiation Sciences, Curtin University, Kent St, Bentley, WA, 6102, Australia

## Abstract

Globally, there has been an increase in the use of herbal remedies including traditional Chinese medicine (TCM). There is a perception that products are natural, safe and effectively regulated, however, regulatory agencies are hampered by a lack of a toolkit to audit ingredient lists, adulterants and constituent active compounds. Here, for the first time, a multidisciplinary approach to assessing the molecular content of 26 TCMs is described. Next generation DNA sequencing is combined with toxicological and heavy metal screening by separation techniques and mass spectrometry (MS) to provide a comprehensive audit. Genetic analysis revealed that 50% of samples contained DNA of undeclared plant or animal taxa, including an endangered species of *Panthera* (snow leopard). In 50% of the TCMs, an undeclared pharmaceutical agent was detected including warfarin, dexamethasone, diclofenac, cyproheptadine and paracetamol. Mass spectrometry revealed heavy metals including arsenic, lead and cadmium, one with a level of arsenic >10 times the acceptable limit. The study showed 92% of the TCMs examined were found to have some form of contamination and/or substitution. This study demonstrates that a combination of molecular methodologies can provide an effective means by which to audit complementary and alternative medicines.

The use of complementary and alternative medicine is becoming increasingly popular worldwide^1^,^2^. It is generally believed that since herbal remedies are of natural origin, they are therefore safe, that there are legal safeguards in place to ensure quality, and that they have fewer side effects than conventional medicines^3^,^4^. Recently, questions surrounding the safety and legality of complementary and alternative medicines (CAMs), such as traditional Chinese medicine (TCM), have prompted increased scrutiny. Several studies have shown that there is a lack of accuracy in labelling of some TCM, with undeclared animals and plants, including toxic and endangered species identified by DNA analyses^5^,^6^,^7^. It has also been shown that some TCM contain unsafe levels of heavy metals^8^, or can be adulterated with pharmaceutical drugs^9^,^10^,^11^. However, the negative health impacts of herbal medicines are difficult to quantify, as reporting rates for adverse reactions are low and the use of herbal medicines is often not reported to healthcare professionals^9^.

Regulatory bodies such as the US Food and Drug Administration (FDA), the UK Medicines and Healthcare Products Regulatory Agency (MHRA) and the Therapeutic Goods Administration (TGA) in Australia are entrusted with regulating herbal products, including TCMs, prepared or sold within their borders. Medicines that are thought to contain low-risk ingredients often only require the manufacturer to declare what is contained within their products, a form of regulation referred to as ‘light-touch’^12^. To assess the effectiveness of this regulatory system, an accurate post-market auditing strategy of herbal medicines is required, a sentiment endorsed in World Health Organisation strategy reports^13^.

The field of complementary and alternative medicine polarises the public, health care professionals, regulators and researchers, but efficacy arguments aside, most would agree that herbal CAMs should be safe, unadulterated, legal and honestly labelled. In this study, we set out to evaluate the content of 26 legally purchased TCMs, using a combination of molecular screening methods. This study is the first of its kind, and aims to provide much-needed pharmacovigilance strategies for pre- and post-market auditing of herbal CAMs.

## Results

### Genetic analyses

The plant and animal composition of the 26 TCMs were genetically analysed using both 454 (Roche) GS Junior and Ion Torrent PGM NGS platforms (Table 1). Multiple mtDNA and plastid genes were targeted for species identification in this study. Of the 26 TCM samples collected, 22 (84%) had sufficient DNA (determined by qPCR) to progress to amplicon sequencing. The raw data that passed quality filter totalled 593,100 reads, averaging at approximately 32,000 reads per sample across the markers.

The taxonomic assignments from the mtDNA and plastid DNA data are listed in Table 1 and discussed below. As the taxonomic assignments of every plant contained in the TCM were too numerous to list here, a sub-set of the plant data is shown in Table 1. The plant family and genera in the table are primarily those that should be flagged on the grounds of toxicity, legality or relate to discussions of the openly acknowledged ingredients. Finally, in Table 1 we have carefully examined the declared plant and animal ingredients (where available) of both TGA listed and unlisted TCMs to evaluate their accuracy. A TCM was deemed to have not correctly declared its ingredients when DNA originating from a ‘concerning’ or trade restricted plant/animal was detected.

### Toxicological data

Each of the 26 TCM samples was extensively tested for potential adulterants using LC-MS analysis. Each of the compounds was carefully assessed relative to known standards, and a limit of quantitation and limit of detection was constructed for each compound. A total of 18 different toxicological compounds were detected in 13 TCMs listed in Table 2. In positive samples, the number of adulterants and/or undeclared substances varied from one (TCMs 8, 13, 22, and 26) to six detected in the TGA-listed TCM2.

The amounts of the adulterants and/or undeclared substances detected were variable between samples as indicated in Table 2. The mere presence of some of these compounds contravenes both TGA regulations and Australian law. In Table 2, the dosage instructions were used with each medicine (see Table S3) and the calculated nominal dosage of the pharmacological adulterant under this regime was compared to doses used in clinical practice.

### Heavy metal data

Twenty-five out of the 26 TCM samples were screened for heavy metal contamination using SF-ICP-MS. The aqueous state of TCM17 prevented it from being analysed using the above method. Three potentially toxic metals to humans, arsenic, cadmium and lead were the focus of this analysis, many of which are present in quantities that exceed recommended daily intakes (see Table 3 for calculations). Of the 25 TCMs analysed, 20 contained at least one of these metals (arsenic, lead or cadmium) in varying quantities (Table 3). Eleven samples contained all three toxic metals with amounts detected ranging from just above the acceptable TGA limit (for a 60 kg person taking the recommended dose) up to and beyond 10 times the TGA limit for medicines (Table 3). As with the toxicological data described above, the exposure estimates to the heavy metals were calculated based on the dosage instructions (Table S3) on the medicine packaging and then referenced to the TGA guidelines for heavy metal concentrations in medicines. The raw SF-ICP-MS results in parts per million (PPM) for all heavy metals screened in this study can be found in Table S4.

**Figure 1:**
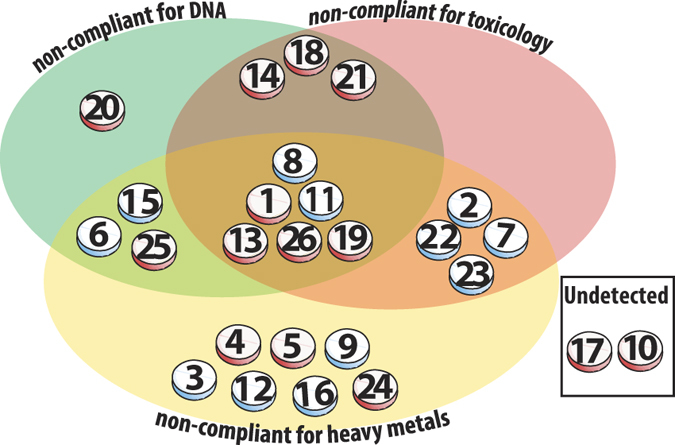
Summary of traditional Chinese medicines (TCMs) tested in this study that contained toxic metals, undeclared or illegal contents as determined by DNA, toxicological, and heavy metal screening methods. Each TCM tested is represented in the diagram as a tablet; blue shading on tablets indicate AUST L listed medicines, red shading are not-listed with the TGA regulatory body. TCMs deemed non-compliant for DNA (green), toxicology (pink) and heavy metals (yellow) or a combination thereof, are represented within the Venn diagram. ‘Non compliance’ is defined as containing an illegal or undeclared species, undeclared pharmaceutical, or heavy metal in quantities beyond the allowable daily dosage limit. Two TCM’s were classified as ‘undetected’ using the testing methods described.

Figure 1 provides a summary of all TCMs that were deemed to be either compliant or non-compliant based on the three screening methods combined. Out of the 26 medicines analysed, two of these (8%) were not found to contain undeclared substances and are therefore displayed as being ‘compliant’ with the regulatory body standards.

## Discussion

This study describes the first detailed combined DNA, toxicological, and heavy metal audit of herbal medicine employing both next generation DNA sequencing technologies and mass spectrometry-based detection. The focus of this study was on 26 TCMs purchased “over the counter” within Australia, however many of these medicines are also for sale on the international market. Of the 26 TCM samples, 12 were classified as “Listed” by the TGA, with the remainder having no listing meaning that they should not have been commercially available. In Australia, and elsewhere, herbal products such as TCMs are regarded as low-risk and therefore undergo less stringent regulation. In some jurisdictions TCMs are simply classified as ‘dietary supplements’. Regardless of how they are categorised, the regulatory framework relies heavily, or exclusively on the assumption that manufacturers are making accurate declarations regarding the composition of their products. In the case of the TCMs studied here, it is clear that such declarations are not always correct; i.e. there were major discrepancies between what was declared, either to the regulatory agency, or to the consumer in the form of an ingredient list.

Coghlan *et al*. (2012) had demonstrated that a DNA-based NGS approach is a powerful way to scrutinise TCM for plant and animal components^5^. The potential for DNA barcoding to be used for improving pharmacovigilance of herbal medicines has also been well highlighted in a recent review^14^. In the current study, toxicological and heavy metal analyses complement the genetic data so that DNA composition, potential adulterants and contaminating heavy metals can be assessed in combination (summarised in Fig. 1). Our analysis of the 26 TCMs identified a complex list of materials that ranged from harmless contaminants, to illegal or potentially dangerous adulterants and/or heavy metals.

The TCM that most clearly breaches international law is TCM8 (used to treat “arthritis” and “pain”) where two mtDNA signatures (differing by one polymorphism) were recovered that provided a 100% match to species within the genus *Panthera*. This sample contained DNA from snow leopard (*Panthera uncia)*, and possibly tiger (*Panthera tigris*), both of which are listed within the Convention on the International Trade in Endangered Species of flora and fauna (CITES) Appendix I. Appendix I affords the highest level of trade restriction. This result was checked using independent DNA isolations from TCM8 and sequencing them on 454, Ion Torrent and Illumina NGS platforms. Finally, the presence of snow leopard was confirmed by successful amplification of *P. uncia* using a species-specific PCR assay^15^. TCMs have previously been shown to contain trade-restricted species listed within the appendices of CITES^16^,^17^,^18^. The presence of *Panthera* DNA in a TGA ‘Listed’ medicine is therefore of great concern. On the basis of this identification alone, TCM8 is illegal to import or sell and an incorrect declaration has been made to the regulatory agency.

Material from taxa such as *Panthera* (TCM8), pit viper (TCM 11) and frogs (TCM 19) are likely to have been deliberately added as a primary ingredient. However the reproducible detection of DNA from ‘domestic’ animals (cow, goat, sheep, dog, cat, and rat, see Table 1) in a given sample could mean that these are either also undeclared ingredients, or alternatively are inadvertent contaminants during manufacture. As none of these products have declared any animal ingredients, this may indicate major deficiencies in manufacturing standards.

The presence of DNA assigning to plant species such as *Asarum* (TCM1) is of concern due to the clear carcinogenic risks associated with aristolochic acid^5^,^19^,^20^. Likewise, the undeclared presence of DNA from the *Ephedra* genus in this same TCM makes the product illegal to sell in most jurisdictions. Four TCM samples contained DNA that could be assigned to the genus *Apocynum*, which has very restricted use and is on the registry of poisonous (FDA), restricted or prohibited plants (MHRA and TGA). *Apocynum* species are known to contain cardiac glycosides (e.g. cymarin), which are cardiotoxins that can cause arrhythmias.

Up to 24% of TCM preparations have been previously reported to be adulterated with pharmaceuticals^11^. The current toxicological data showed between 19% and 50% of the preparations were adulterated depending on whether compounds such as ephedrine and salicylic acid were naturally derived or were synthetic adulterants.

From a toxicological perspective, TCM2 (described as “reducing hay fever” and “nasal secretions”) was perhaps the most concerning as a ‘cocktail’ of six undeclared compounds were detected: ephedrine (1097 μg/g), chlorpheniramine (1025 μg/g), salicylic acid (25.2 μg/g), amoxicillin (18 μg/g), methylephedrine (10 μg/g) and small quantities of warfarin (4 μg/g). The interactions of the analgesic, antibiotic, stimulant and antihistamine drugs in this single TCM preparation are difficult to predict. This may be even more significant if the preparation is used by children or pregnant women.

In some instances there was a clear correlation between the genetic and toxicological analyses. The ephedrine detected in TCM1 correlates with the detection of *Ephedra* DNA. In other instances *Ephedra* DNA could not be detected when ephedrine was present, and this may be because a synthetic adulterant was added to the preparation or, alternatively, the DNA was too highly degraded for detection. In another example, there was no obvious genetic sequence that could account for the brucine (3405 μg/g), and strychnine (1509 μg/g) detected in TCM1, although species outside of the *Strychnos* genus may also generate these alkaloids.

A potentially recurring theme was that the adulterant compounds found in the TCMs were often ‘tailored’ to deliver the purported therapeutic outcome^21^. For example, TCM18 which aimed at enhancing weight gain with appetite stimulation contained both dexamethasone (6034 μg/g) and cyproheptadine (20333 μg/g), the latter of which is a known appetite enhancer^22^. The dose of cyproheptadine in a single capsule was 8mg, while the typical therapeutic dose is 2–4 mg. TCM18 also contained DNA from the genus *Morus* (mulberry, Table 1), which some practitioners claim, plays a role in digestive function. Such findings are not only of concern to the consumer, but also flag the need for detailed auditing of herbal preparations prior to evaluation in clinical trials. It is possible, indeed likely, that given the scope and dose of the adulterants (listed in Table 2) that many TCM’s would perform better than placebo in randomised trials.

Of the 16 heavy metals screened for in this study, particular attention is paid to three toxic metals; arsenic, cadmium and lead. All three metals are highly toxic to humans if exposure is high, potentially causing severe adverse health effects or death, with arsenic and cadmium also having documented carcinogenicity^23^,^24^. TCM19 stands out as one of the most highly toxic medicines upon heavy metal analysis, with the levels of arsenic detected well over 10 times the daily limit for medicines (Table 3). Coupled with this finding is also the detection of a synthetic drug, diclofenac, and multiple undeclared animal DNA signatures found within this sample. TCM1 is again highlighted with levels of lead and cadmium detected that were over two times the TGA limit for medicines, as well as multiple pharmaceutical adulterants found, and potentially toxic plant material. Both TCM1 and TCM19 are non-listed medicines that again should not have been available for sale within Australia.

This study presents genetic, toxicological, and heavy metal data that should be of serious concern to regulatory agencies, medical professionals and the public who choose to adopt TCM as a treatment option. Of the 26 TCMs investigated, all but two can be classified as non-compliant on the grounds of DNA, toxicology and heavy metals, or a combination thereof (Fig. 1). In total, 92% were deemed non-compliant with some medicines posing a serious health risk. The multi-tiered approach outlined in this study provides a much-needed auditing toolkit that should swiftly form the basis of best-practice pharmacovigilance across the CAM sector.

## Methods

### Sample collection

Twenty-six pre-packaged TCM samples were purchased from retail stores and TCM practitioners in South Australia, many of which are also available for purchase internationally online. Sample types included; capsules, tablets, and herbal teas and one liquid sample. A list of the samples (with branding anonymised), including whether the product is categorised as a listed medicine by the regulatory body (the TGA), can be found in the supplementary information (Table S1).

### DNA extraction and quantification

DNA characterisation of the TCM samples followed Coghlan, Haile *et al*. (2012)^5^. Briefly, small amounts of each sample (between 45–300 mg), together with extraction controls, were digested overnight, on a shaking heat block at 55 °C, in 0.7–1.5 mL of tissue digest buffer consisting of; proteinase K powder (Amresco, OH, USA), 20 mM Tris (Sigma, MO, USA), 2.5 mM EDTA (Invitrogen, CA, USA), 5 mM CaCl_2_ (Sigma), 20 mM dithiothreitol solution (Thermo Fisher Scientific, MA, USA), 1% sodium dodecyl sulfate (Invitrogen), and Milli-Q water. 200 μL of digest was mixed with 1 mL of Qiagen (CA, USA) PB buffer and purified and washed on a silica spin column and finally eluted with 50 μL of EB buffer (Qiagen, CA, USA).

The extracted DNA was quantified by amplifying plant and mammal DNA gene regions: *trn*L g/h, *rbc*L, and 16S rRNA^25^,^26^,^27^, (primer information can be found in Supplementary Table S2) using an ABI StepONE qPCR (Applied Biosystems, USA) platform. All PCRs were carried out in a 25 μL volume including: 2 mM MgCl_2_ (Applied Biosystems, USA), 1 × Taq polymerase buffer (Applied Biosystems, USA), 0.4 μM dNTPs (Astral Scientific, Australia), 0.1 mg bovine serum albumin (Fisher Biotec, Australia) when required to counteract inhibition, 0.4 μM of each primer, 0.2 μL of AmpliTaq Gold DNA polymerase (Applied Biosystems, USA) and SYBR-Green dye. The qPCR conditions were: 50 cycles of 95 °C for 30 seconds, annealing at primer specific temperature (Supplementary Information, Table S2) for 30 seconds, 72 °C for 30 seconds. The qPCR was carried out for each sample at three dilution points (undiluted, 1/10 and 1/100) to gauge template copy number and identify if PCR inhibitors were present.

### Amplicon generation

Fusion primers with unique 6–8 bp multiplex identifier (MID) tags were designed for the plant and mammal primer sets used for qPCR above. Amplicon sequencing was carried out on both a 454 (Roche, USA) GS Junior instrument as described previously^5^, and using a Personal Genome Machine (PGM) (Life Technologies, CA, USA) according to manufacturer’s protocols. All amplicons were generated in triplicate and assigned unique forward and reverse multiplex identifier (MID) tags to ensure that any contamination from previously generated amplicons could be excluded post sequencing. Primer sequences used in this study are provided in the supplementary information (Table S2).

### Bioinformatic analysis

The sequencing output files were retrieved, filtered, and processed using Geneious (v6.1)^28^. Reads were deemed to have passed filter when they had exact matches for primers and MID tag sequences at both amplicon ends. A non-redundant sequence set (removal of exact matches) for each sample was generated using USEARCH^29^. These files were then analysed for chimeras in USEARH using the UCHIME *de novo* method^30^ and removed, along with singletons and imported into YABI^31^ where a BLASTn search was conducted against the National Centre for Biotechnology Information (NCBI) GenBank NR database^32^,^33^. The resultant BLAST files were then imported into the program MEtaGenome ANalyzer (MEGAN v4.69.4)^34^ for taxonomic scrutiny. The amplicon sequences were assigned using the following lowest common ancestor parameters: min score of 65, top percent of 5, and min support of 1. Each taxonomic assignment was critically evaluated using available databases and is a conservative estimate of families, genera and species. Processed amplicon data described herein is available from Dryad Digital Repository: http://dx.doi.org/10.5061/dryad.ps04c.

### Toxicological analysis

TCM samples were crushed in a clean mortar and pestle, and 5 mg of each was extracted with methanol containing 0.05 mg/mL diphenhydramine (internal standard), for 1 minute at 6500 rpm in a Precellys tissue lyser (Bertin Technologies, France). Extracts were centrifuged for 5 minutes at 1.3 × 10^4^ × *g* and diluted 1:10 with LC-MS water for analysis. The final internal standard concentration was 5 ng/mL.

Samples were analysed using a Varian 212 HPLC system coupled to a 325-MS triple quadrupole mass spectrometer, using electrospray ionisation (Agilent Technologies, USA). The column was a Restek Ultra Aqueous C_18_ (100 mm × 2·1 mm × 3 μm) (Restek Technologies, USA), with mobile phase A being LC-MS water and mobile phase B being 0.1% formic acid in acetonitrile. Analysis was repeated with mobile phase B as methanol to expand coverage of possible adulterants. The nebuliser gas (air) was set at 70 psi, the vortex gas at 30 psi and 300°C and the drying gas at 25 psi and 200°C (both N_2_). Mobile phase was held at 95% A for 2 minutes before ramping to 100% B by 9 minutes, held for 5 minutes, back to 95% A by 15 minutes, held for 5 minutes.

Eighteen likely adulterants were selected and, to establish optimal MS/MS conditions, standards of each were infused into the MS at 1 μg/mL. A minimum of two transitions per compound were selected and the dwell time for each transition was 0.025 s for a total scan time of 0.85 s, with the detector set at 1100 V. Standard curves were generated for each compound over the range 0.1 to 100 ng/ml. Data was analysed using Varian MS Workstation v6.9.3 (Agilent Technologies), with peak areas normalised to the internal standard.

### Heavy metal analysis

TCM samples were leached in concentrated nitric acid (HNO_3_) to screen for bioavailable toxic metals. The “acid leachable” method does not determine total metal concentrations but is representative of metals released from the TCM samples *in-vitro*. For reporting purposes, the concentrations determined by the method should be considered an underestimate of the total metal concentrations contained within TCM. Powdered TCM samples (~0.3 g) were added to acid-cleaned polypropylene vials containing 5 mL of ultrapure nitric acid (~70% concentration, double distilled) and leached for 24 hours at room temperature. Following acid leaching samples were made up to 10 mL with ultrapure water (>18 mΩ), filtered (acid-cleaned 0.2 μm PTFE filter membrane) and diluted 1: 75 with 3% ultrapure HNO_3_.

Sixteen heavy metals were determined under ultra-trace clean conditions using a sector-field inductively coupled plasma mass spectrometer (SF-ICP-MS, Thermo Fisher Scientific ELEMENT XR) housed in the Curtin University ultra-clean TRACE facility. Samples were mixed with an internal standard (Indium) and introduced to the SF-ICP-MS using an auto-sampler (Elemental Scientific Inc, Seafast II) enclosed in a metal free hood. Instrumental and full procedural blanks (3% HNO_3_), replicates and quality control standards (high Purity Standards, CWW-TM-A) were determined at regular intervals throughout the analysis. Internal standard normalised ions intensities were quantified using “matrix matched” external standards prepared by serial dilution from commercial primary standards. The results were blank subtracted and corrected for dilution. TCMs were deemed non-compliant if the concentration of the heavy metals lead, cadmium and arsenic were above the threshold daily intake levels set by the TGA for a 60kg person.

## Additional Information

**How to cite this article**: Coghlan, M. L. *et al*. Combined DNA, toxicological and heavy metal analyses provides an auditing toolkit to improve pharmacovigilance of traditional Chinese medicine (TCM). *Sci. Rep*. **5**, 17475; doi: 10.1038/srep17475 (2015).

## Supplementary Material

Supplementary Information

## Figures and Tables

**Table 1:** Selected plant and animal families and genera detected in 18 traditional Chinese medicine (TCM) samples by mitochondrial and plastid DNA analysis.

	Family	Genus	AUST L TCMs							Non Listed TCMs										
			6	7	8	9	11	15	23	1	4	13	14	18	19	20	21	24	25	26
			37,196 reads	30,292 reads	23,904 reads	15,892 reads	28,785 reads	22,514 reads	31,822 reads	18,333 reads	27,277 reads	17,935 reads	23,450 reads	31,796 reads	33,877 reads	24,521 reads	42,796 reads	25,083 reads	25,751 reads	36,002 reads
Plant Content	Acanthaceae	*Andrographis*											✓		✓				✓	
	Amaranthaceae	*Amaranthus*			✓															
	Apiaceae	*Bupleurum*		✓																✓
	Apocynaceae	*Apocynum*	✓					✓				✓								✓
	Asparagaceae	*Anemarrhena*			✓														✓	
	Asteraceae		✓						✓	✓	✓			✓		✓	✓		✓	✓
	Araliaceae								✓	✓						✓				✓
	Aristolochiaceae	*Asarum*								✓										
	Campanulaceae	*Codonopsis*	✓																	
	Cupressaceae	*Platycladus*																	✓	
	Fabaceae	*Astragalus*	✓		✓	✓				✓								✓	✓	✓
		*Glycyrrhiza*	✓	✓		✓		✓		✓		✓			✓			✓	✓	✓
	Dipsacaceae	*Dipsacus*			✓					✓										
	Ephedraceae	*Ephedra*								✓										
	Lamiaceae	*Mentha*	✓							✓	✓								✓	
		*Perilla*					✓							✓					✓	✓
		*Scutellaria*					✓													
	Lardizabalaceae	*Sargentodoxa*								✓										
	Loranthaceae	*Taxillus*								✓		✓								
	Moraceae	*Morus*					✓			✓		✓		✓					✓	✓
	Nelumbonaceae	*Nelumbo*		✓					✓				✓							
	Pedaliaceae																			✓
	Polygonaceae	*Oxyria*			✓					✓										
	Paeoniaceae	*Paeonia*			✓			✓	✓	✓			✓							
	Salicaceae	*Salix*		✓																
	Rosaceae	*Prunus*	✓							✓						✓				
	Rutaceae	*Citrus*					✓													
		*Phellodendron*			✓															
	Zingiberaceae		✓																	
Animal Content	Bovidae	*Bos*												✓	✓	✓	✓			✓
		*Bubalus*													✓					
		*Capra*												✓						
		*Ovis*								✓										
	Canidae	*Canis*			✓		✓							✓	✓					
	Felidae	*Panthera*			✓															
		*Felis*													✓				✓	
	Muridae	*Rattus*					✓						✓						✓	
	Rhacophoridae	*Polypedates*													✓					
	Viperidae	*Deinagkistrodon*					✓													

**Table 2:**
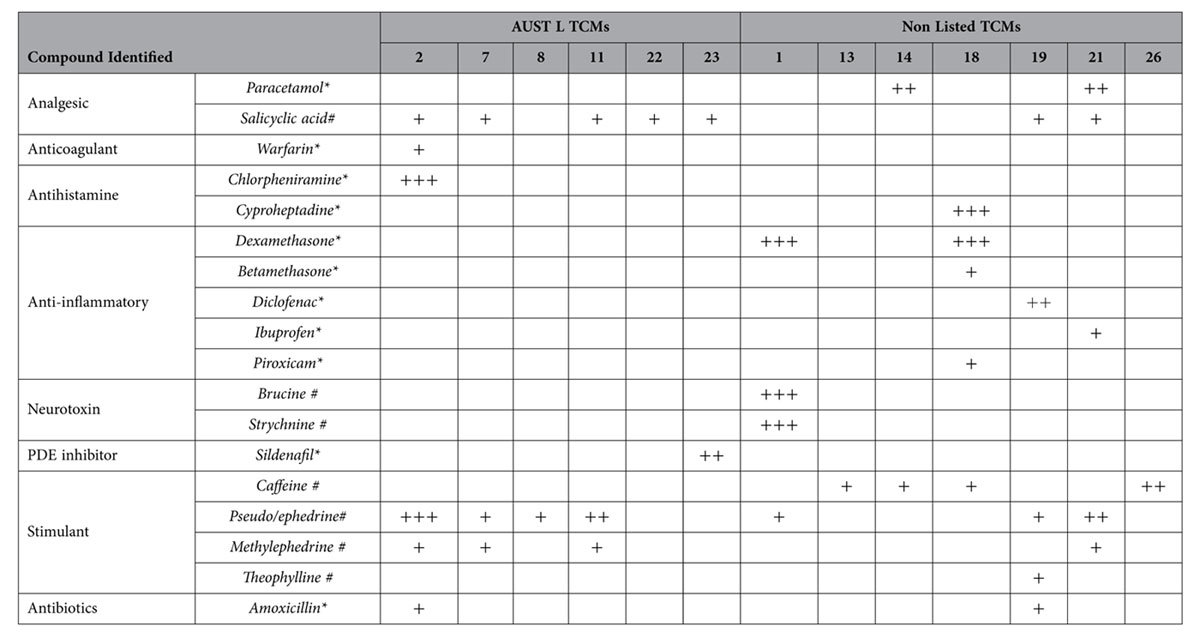
Adulterants/undeclared compounds detected in the traditional Chinese medicine (TCM) samples tested in this study.

Of the 26 TCMs screened using toxicological/metabolomic techniques, those appearing in the table below were found to contain pharmaceutical and/or naturally occurring compounds, none of which were on ingredients lists.

+Trace amount detected.

++Amount detected is physiologically relevant, above trace levels but below clinically used levels.

+++Clinically significant amount detected.

^*^Synthetically derived compound.

^#^Could be naturally occurring or synthetically derived.

**Table 3:** Toxic heavy metals detected in the traditional Chinese medicine (TCM) samples tested in this study.

AUST L TCMs	Heavy Metal Detected		
	Arsenic	Lead	Cadmium
2	**++**	**++**	**++**
3	**+**		**++**
6	**+**		**+**
7	**++**	**++**	**++**
8		**+**	**++**
9	**+**		**+**
11	**++**	**++**	**++**
12	**++**	**++**	**++**
15	**+++**		**+**
22	**+**		
23	**+**	**+**	**++**
**Non Listed TCMs**			
1	**+**	**++**	**++**
4	**+++**	**+++**	**++**
5		**++**	**++**
13	**++**	**+++**	**+++**
16	**++**		**+**
19	**++++**	**++**	**+**
24		**++**	**++**
25	**++**	**++**	**+++**
26	**++**	**++**	**+++**

Of the 25 TCMs screened using SF-ICP-MS, those appearing in the table below were found to contain the toxic metals arsenic, lead or cadmium in varying quantities.

**+** At or less than two times the TGA limit for medicines (based on 60 kg person; 1.2 μg/daily dose arsenic, 2.2 μg/daily dose lead, 0.22 μg/daily dose cadmium). **++** Greater than two times the TGA limit for medicines. **+++** Greater than 10 times the TGA limit for medicines. **++++** Much greater than 10 times the TGA limit for medicines.
