# Supplementary material for: Combined DNA, toxicological and heavy metal analyses provides an auditing toolkit to improve pharmacovigilance of traditional Chinese medicine (TCM)
Source: Sci Rep. 2015 Dec 10;5:17475. doi: 10.1038/srep17475 (PMC4675079; doi:10.1038/srep17475)
Supplement: Supplementary Information [file srep17475-s1.pdf]

Combined DNA, toxicological and heavy metal analyses provides an auditing toolkit to improve pharmacovigilance of traditional Chinese medicine (TCM).

Megan L. Coghlan[1], Garth Maker[2,3], Elly Crighton[2,3], James Haile[1], Dáithí C. Murray[1], Nicole E. White[1], Roger W. Byard[4,5], Matthew I. Bellgard[6], Ian Mullaney[2,3], Robert Trengove[2], Richard J.N. Allcock[7,8], Christine Nash[5], Claire Hoban[4], Kevin Jarrett[9], Ross Edwards[9], Ian F. Musgrave[4] and Michael Bunce[1*].

Table S1 TCM products purchased for this study from Adelaide, South Australia, including sample ID, medicinal type and listed package ingredients.

Sample ID	Medicinal type	AUSTL number	Ingredients listed on packaging
TCM 1	Capsules	No	None listed
TCM 2	Tablets	Yes	Scutellaria baicalensis root, Xanthium sibiricum seed, Magnolia liliflora flower bud, Lonicera japonica flower, Atractylodes macrocephela root, Mentha haplocalyx leaf, Angelica dahurica root
TCM 3	Tablets	Yes	Xanthium sibiricum seed, Angelica dahurica root, Platycodon grandiflorum root, Magnolia liliflora flower bud, Schizonepeta tonuifolia herb, Ledebouriella divaricata root, Chrysanthemum sinense flower, Anemarrhena asphodel lids fruit, Forsythia suspensa fruit, Shizandra chinensis
TCM 4	Pills	No	None listed
TCM 5	Pills	No	Radix notoginseng, Corex eucommiae, Radix ginseng, Ginamomum cassia presl, Olibanam, Herba taxilli, Fructose schisandrae, Radix angelicae pubescentis
TCM 6	Pills	Yes	Herbal powder, Angelica polymorpha root, Codonpsis pilosura root, Polygonatum sibirica rhizome, Polygamy sibirica root, Atractylodes macrocephela rhizome, Poria cocos fruit body, Zizyphus jujube fruit, Glycyrrhiza urlensis root, Fructose, glucose, sucrose (from honey)
TCM 7	Pills	Yes	Herbal powder, Codonopsis pilosura root, Astragalus membranaceus root, Paeonia lactiflora root, Atractylodes macrocephela rhizome, Poria cocos fruit body, Rehmannia glutinosa root, Angelica polymorpha root, Cinnamomum cassia branch, Glycyrrhiza uralensis root
TCM 8	Pills	No	Achryanthes bidentata root, Anemarrhena asphodeloids root, Angelica polymorpha root, Angelica pubenscens root, Astragalus membranaceus root, Atractylodes macrocephela root, Chaenomeles specious fruit, Cuscuta hygrophilae seed, Cynomorium songariorum stern, Dipsacus asper root, Eucommia ulmoides stern bark, Gentians macrophylla root, Ledebouriella divaricata root, Lycium barbarym fruit, Notoperyguim incisium root, Paenoia lactiflora root, Panax ginseng root, Phellodendron amurense stem bark, Poria cocos root, Psoralea coryliliflora fruit, Rhemmania glutinosa root, Sugars from honey
TCM 9	Pills	Yes	Herbal powder, Astragalus membranaceus, Glycyrrhiza uralensis root and rhizome, Codonopsis pilosula root, Atractylodes macrophala rhizome, Angelica polymorpha root, Citrus reiculata frui peri, Cimifuga heracleifolia root, Ziziphus jujuba fruit, Zingiber offinale rhizome, Glucose, sucrose from licorice
TCM 10	Flakes	No	None listed
TCM 11	Tea bags	No	Eritrea japonica leaf, Angelica dahurica root, Fritillaries thundbergii bulb, Peucedanum praeruptorum root, Xanthium sibiricum fruit, Scutellaria baicalensis root, Pinellia ternata tuber, Glycyrrhiza uralensis root, Cynanchum stauntonii root, rhizome, Ephedra sinica stem, Morus alba root skin, Perilla frutesciens leaf, Poria cocos fruit, Citrus reticulata fruit peri.
TCM 12	Capsules	Yes	Zizyphus spinosa fruit flesh, Poria cocos root, Polygonum multiflorum stem, Schisandra chinensis fruit, Polygala sibirica root
TCM 13	Tea bags	No	Radix gentiane macrophyllae, Cortex eucommiae, Radix polygoni sue multiflori, Rhizome ligustici, Radix notoginseng, Epimedium grandiflora, Spatholobus suberectus, Radix astragati seu hedysari, Fructus lycii, Fructus rubi, Semen cuscutae, Rosae cuscutae, Fructus mori, Fructus comi, Fructus hordei germinatns, Clematis chinensis, Radix rehmanniue, Nelumbo nucifera, Poria, Rhizoma dioscoreae, Semen sesami, Ramulus loranthi, Radix glycyrrhizae
TCM 14	Pills	No	None listed

Table S1 continued

Sample ID	Medicinal type	AUSTL number	Ingredients listed on packaging
TCM 15	Pills	Yes	Zizyphus jujuba fruit, Poria cocos fruit body, Paeonia lactiflora root, Polygonum multiforum tuber, Glycyrrhiza uralensis root, rhizome, Ophiopogon japonicus tuber, Biota orientalis seed, Curcuma aromatica tuber, Pimellia ternata rhizome, Chrysanthemum indicum flower, Polygala sibirica root, Schizandra chinensis fruit, Zizyphus spinosa seed
TCM 16	Pills	No	Herbal powder, Rehmannia glutinosa root, Dioscorea opposita rhizome, Cornus offcinalsis fruit flesh, Paeonia suffruiticosa root bark, Portia cocos fruit body, Alisma orientale tuber
TCM 17	Drink	No	None listed
TCM 18	Capsules	No	None listed
TCM 19	Capsules	No	None listed
TCM 20	Capsules	No	Cordyceps, Ganoderma
TCM 21	Capsules	No	None listed
TCM 22	Capsules	Yes	Zizyphus jujuba seed, Anemarrhena asphodeloides rhizome, Salvia miltiorrhiza root and rhizome, Polygala sibirica root, Biota orientalis seed, Atractylodes macrocephela rhizome, Glycyrrhiza uralensis root and rhizome, Panax ginseng root and rhizome, Ophipogon japonicus root tuber, Schizandra chinensis fruit, Morus alba fruit, Poria cocos white fruit
TCM 23	Pills	Yes	Paeonia lactiflora root, Ligusticum wallichii root, Carthamus tinctorius flower, Poria cocos root, Sitesbeckia orientalis whole plant, Notopterygium incisium root, Lonicera japonica flower, Citrus reticulata peel, Angelica polymorpha root, Rehmannia glutinosa root, Atractylodes lancea root, Achyranthes bidentata root, Ledebouriella seseloides root, Atractylodes macrocephela rhizome, Glycyrrhiza uralensis root
TCM 24	Tea bags	No	Astragalus membranaceus, Ophiopogon, Poria cocos, Licorice, Cornus officinalissieber, Reb tea
TCM 25	Tea bags	No	Fructus schinsandrae, Caulks spatnolobi, Rhizoma anemarrgenae, Rhizoma ligustici chuanxiong, Rhizoma acori graminei, Radix polygoni multiflori, Radix astragati seu hedysari, Fructus lycii, Fructus mori, Radix adenophorae strictae, Radix regmanniue, Polygonum multiflorum, Rhizoma polygonal odorati, Semen raphani, Fructus hordei germaniatus, Flos lonicerae, Herba exliptae, Rhizoma alismatis, Nelumbo nucifera, Semen ziziphi spinosae, Poria, Semen sesami, Radix glycyrrhizae
TCM 26	Tea bags	No	Fructus xanhii, Radix angelicae dahuricae, Flos magnoliae, Rhizoma ligustici chuanxiong, Herba menthae, Radix astragali seu hedysari, Rhizoma anemarrhenae, Fructus hordei germinatus, Semen cassiae, Semen raphani, Radix alismatis, Folium mori, Flos lonicerae, Bulbus fritillariae thunbergii, Nelumbo nucifera, Poria, Semen sesami, Rhizoma dioscoreae, Radix glycyrrhixsae

Table S2. List of primers used in this study for generating fusion tagged amplicons for next generation sequencing (NGS)

Primer Name	Target	Primer Sequence (5'-3')	Annealing temp.	Product size	Source
Titanium Primer A	Sequencing primer	CGTATCGCCTCCCTCGCGCCATCAG		N/A	(Roche)
Titanium Primer B		CTATGCGCCTTGCCAGCCCGCTCAG	N/A		
Ion Torrent A	Sequencing primer	CCATCTCATCCCTGCGTGTCTCCGACTCAG		N/A	(Life Technologies)
Ion Torrent P1		CCTCTCTATGGGCAGTCGGTGAT	N/A		
trnL g	Plant species	GGGCAATCCTGAGCCAA		50-70 bp	(Taberlet, Coissac *et al.* 2007)
trnL h		CCATTGAGTCTCTGCACCTATC	52 °C		
rbcL h1aF	Plant species	GGCAGCATTCCGAGTAACTCCTC		80-100 bp	(Poinar, Hofreiter *et al.* 1998)
rbcL h2aR		CGTCCTTTGTAACGATCAAG	52 °C		
16S mam1	Mammalian species	CGGTTGGGGTGACCTCGGA		90-100 bp	(Taylor 1996)
16S mam2		GCTGTTATCCCTAGGGTAACT	57 °C		

Individual fusion primers consisted of a forward or reverse sequencing primer followed by a 6-8 bp multiplex identifier (MID) tag, and one of the above gene specific primer sequences.

Table S3. Dosage instructions and sample weights of the 26 traditional Chinese medicines (TCMs) tested in this study.

**Sample ID**	**Dose**	**Weight (g)**
TCM1	2-3 capsules, 2 times daily	0.527
TCM2	3-5 tablets, 3 times a day	0.366
TCM3	3-4 tablets, 3 times daily	0.559
TCM4	2 bottles are to be taken once every two hours, 4 times daily. 1/2 a bottle for children. (1 bottle contains about 2.3g of pills)	0.029
TCM5	4-5 pills, 4 times daily with warm water	0.138
TCM6	8 pills, 3 times daily, half to one hour before meals with warm water	0.164
TCM7	8 pills, 3 times daily, half to one hour before meals with warm water	0.168
TCM8	30- 60 pills each time, 2 times a day with warm or light salty water	0.101
TCM9	8 pills, 3 times daily, half to one hour before meals with warm water	0.191
TCM10	From the internet: one 10g sachet made into a tea	N/A
TCM11	Add 1 to 2 tea bags into 150ml boiled water. Leave for 5 min and then drink. Drink 2 to 3 times a day.	3.23
TCM12	3 capsules, 3 times daily	0.592
TCM13	4 tea bags in 1 litre of water. Boil for 5 minutes	4.45
TCM14	Once a day, 1-2 capsules each time. To be taken just before going to bed, with cool boiled water	0.261
TCM15	15 pills, 3 times daily, half to one hour before meals with warm water	0.181
TCM16	8 pills, 3 times daily, half to one hour before meals with warm water	0.182
TCM17	No English translation	N/A

TCM18	1-2 capsules each time. Take before sleep.	0.378
TCM19	1-2 capsules every night before sleep	0.313
TCM20	1 capsule, 2 times a day or take as often as needed. (Maximum: 2-4 capsules per day)	0.559
TCM21	No English translation	0.447
TCM22	3-4 capsules, 2 a day. If necessary, 1 or 2 capsules more before sleep.	0.344
TCM23	8 pills, 3 times daily, 30 min or 1 hour away from meals with 250ml of water	0.192
TCM24	4 tea bags in 1 litre of water. Boil for 5 minutes	4.54
TCM25	4 tea bags in 1 litre of water. Boil for 5 minutes	4.73
TCM26	4 tea bags in 1 litre of water. Boil for 5 minutes	4.55

Table S4. Heavy metal results from SF-ICP-MS analysis of 25 traditional Chinese medicines (TCMs) tested in this study

Sample ID	Be (ppm)	Sr (ppm)	Mo (ppm)	Ag (ppm)	Cd (ppm)	Sb (ppm)	Tl (ppm)	Pb (ppm)	Li (ppm)	B (ppm)	Na (ppm)	Mg (ppm)	Al (ppm)	P (ppm)	Ca (ppm)	V (ppm)	Cr (ppm)	Mn (ppm)	Fe (ppm)	Co (ppm)	Ni (ppm)	Cu (ppm)	Zn (ppm)	Ba (ppm)	Sn (ppm)	K (ppm)	As (ppm)
TCM23 (1)	8.004E-02	9.305E+02	5.061E+00	6.646E-02	3.074E+00	1.104E+00	4.182E-01	1.362E+01	9.807E-01	5.319E+02	2.372E+04	2.941E+04	1.862E+03	3.326E+04	4.238E+04	6.758E+00	2.612E+01	7.295E+02	2.955E+04	8.903E+00	4.745E+01	1.199E+02	3.757E+02	2.084E+04	2.740E+00	2.363E+05	1.054E+01
TCM11 (2)	9.279E-02	1.852E+03	1.687E+01	3.327E-01	1.907E+00	2.133E+00	8.404E-01	1.601E+02	3.460E+00	9.862E+02	1.341E+04	1.096E+05	4.291E+03	4.273E+04	2.807E+05	1.312E+01	1.514E+01	1.639E+03	6.038E+03	1.847E+01	4.784E+01	6.537E+02	7.231E+02	1.075E+03	7.579E+00	3.694E+05	1.641E+01
TCM26 (3)	2.710E-01	1.223E+03	1.257E+01	1.833E-01	6.787E+00	1.519E+00	6.284E-01	3.469E+01	B.D.L.	8.184E+02	1.449E+04	1.565E+05	7.117E+03	9.427E+04	1.715E+05	1.067E+01	1.933E+01	4.151E+03	5.491E+03	1.091E+01	2.686E+01	4.177E+02	1.043E+03	8.760E+02	3.941E+00	3.219E+05	1.415E+01
TCM8 (4)	4.821E-02	1.148E+03	1.304E+01	3.019E-02	2.672E+00	7.522E-01	2.096E-01	2.105E+01	1.580E+00	4.701E+02	5.945E+04	3.499E+04	4.594E+03	1.916E+05	2.560E+05	1.072E+01	1.902E+01	6.349E+02	6.441E+03	6.632E+00	7.042E+01	1.395E+02	6.058E+02	5.849E+02	1.806E+00	2.110E+05	4.899E+00
TCM24 (5)	B.D.L.	6.019E+02	2.569E+01	4.898E-02	1.450E+00	7.128E-01	4.731E-02	1.062E+01	B.D.L.	5.117E+02	4.718E+03	3.119E+04	8.298E+02	6.254E+04	3.434E+04	2.021E+00	4.185E+00	2.761E+02	1.535E+03	2.229E+00	2.331E+00	1.326E+02	4.692E+02	7.469E+01	3.056E+00	1.958E+05	3.989E+00
TCM1 (6)	3.693E-01	1.257E+03	6.422E+00	1.155E-01	3.174E+00	1.569E+00	2.658E-01	4.557E+01	6.294E+01	6.238E+02	7.972E+04	1.225E+05	5.315E+03	3.253E+04	1.285E+05	1.613E+01	1.202E+01	1.807E+03	8.217E+03	6.879E+00	6.738E+01	1.925E+02	4.662E+02	1.125E+03	3.627E+00	1.940E+05	9.340E+00
TCM4 (7)	B.D.L.	5.541E+02	8.757E+00	1.969E-01	4.278E+00	1.432E+00	4.103E-01	5.464E+01	B.D.L.	4.790E+02	3.421E+03	3.667E+04	3.764E+03	4.514E+04	6.744E+04	9.189E+00	2.065E+01	2.006E+03	1.322E+04	1.041E+01	4.767E+01	2.472E+02	5.679E+02	4.996E+02	2.897E+00	2.209E+05	1.926E+01
TCM14 (8)	B.D.L.	2.194E+01	2.611E+00	B.D.L.	B.D.L.	4.338E-01	B.D.L.	1.053E+00	B.D.L.	2.436E+02	2.429E+03	2.278E+03	1.058E+02	4.481E+03	6.399E+03	B.D.L.	1.678E+00	3.909E+01	1.591E+02	3.808E-02	B.D.L.	2.083E+00	4.958E+01	1.647E+01	1.221E+00	3.059E+03	2.003E-01
TCM12 (9)	1.334E+00	4.926E+02	7.357E+00	4.588E-01	2.192E+00	1.629E+00	2.066E+00	2.736E+01	5.783E+00	1.220E+03	4.848E+04	9.433E+04	1.199E+04	1.024E+05	4.767E+04	2.126E+01	3.131E+01	2.748E+03	1.958E+04	2.437E+01	8.368E+01	1.535E+02	7.174E+02	4.923E+02	6.315E+00	7.799E+05	4.028E+01
TCM3 (10)	7.162E-02	2.104E+02	5.319E+00	B.D.L.	2.693E+00	1.728E+00	3.667E-01	7.431E+00	B.D.L.	6.564E+02	1.645E+04	5.566E+04	3.841E+03	5.265E+04	3.934E+04	7.311E+00	1.962E+01	1.014E+03	3.320E+03	4.802E+00	6.511E+01	6.252E+01	2.648E+02	1.190E+02	1.951E+00	3.676E+05	8.593E+00
TCM5 (11)	7.243E-02	1.181E+03	9.246E+00	2.349E-01	5.834E+00	1.709E+00	5.229E-01	6.167E+01	B.D.L.	5.931E+02	1.337E+04	4.697E+04	3.942E+03	5.220E+04	1.015E+05	1.195E+01	1.193E+01	1.831E+03	4.599E+03	8.316E+00	3.921E+02	1.676E+02	5.136E+02	6.015E+03	1.714E+00	2.548E+05	8.090E+00
TCM21 (12)	1.130E+00	7.216E+02	1.969E+01	1.461E-01	3.223E+01	4.236E+00	2.153E+00	3.363E+01	4.664E-01	1.291E+03	4.357E+04	1.565E+05	1.620E+05	8.786E+04	1.775E+05	1.980E+01	2.781E+01	5.249E+03	6.148E+03	9.548E+01	2.611E+02	1.478E+02	7.676E+02	3.560E+02	3.959E+00	1.089E+06	5.928E+01
TCM18 (13)	1.013E+00	3.089E+03	1.367E+01	4.589E-01	2.403E+00	1.535E+00	3.170E-01	6.724E+01	7.672E+00	1.485E+03	8.203E+03	1.941E+05	7.253E+03	9.237E+04	3.359E+05	1.934E+01	2.062E+01	2.895E+03	1.116E+04	1.012E+01	3.331E+01	2.799E+02	1.148E+03	2.236E+03	3.868E+00	6.845E+05	2.237E+01
TCM16 (14)	B.D.L.	4.979E+02	1.199E+01	6.612E-02	1.647E+00	1.734E+00	5.551E-01	1.066E+01	1.205E+01	5.781E+02	2.605E+04	4.081E+04	3.434E+03	4.827E+04	5.070E+04	9.318E+00	1.666E+01	1.051E+03	1.034E+04	9.525E+00	1.261E+02	1.213E+02	4.465E+02	1.794E+02	4.626E+00	4.044E+05	1.698E+01
TCM20 (15)	B.D.L.	2.630E+02	1.878E+01	7.939E-02	6.591E-01	1.309E+00	B.D.L.	4.304E+00	B.D.L.	5.897E+02	6.040E+03	3.663E+04	1.625E+03	2.223E+05	3.364E+04	2.887E+00	7.606E+00	7.108E+02	4.310E+03	3.479E+00	1.638E+02	2.129E+02	7.873E+02	1.541E+02	4.093E+00	3.918E+05	8.949E+00
TCM9 (16)	3.870E-01	1.883E+03	3.440E+01	2.679E-01	1.084E+00	1.942E+00	5.647E-01	1.108E+01	1.848E+01	1.035E+03	3.262E+04	8.302E+04	5.169E+03	1.163E+05	8.848E+04	2.246E+01	3.443E+01	1.032E+03	8.992E+03	1.100E+01	7.650E+01	2.205E+02	8.182E+02	1.108E+03	4.491E+00	5.079E+05	1.299E+01
TCM2 (17)	1.170E-01	1.088E+03	4.568E+01	2.088E-01	2.867E+00	1.989E+00	1.101E+00	3.547E+01	1.509E+01	1.304E+03	1.072E+05	1.254E+05	2.473E+04	1.196E+05	9.436E+04	1.351E+01	1.947E+01	1.984E+03	7.012E+03	1.378E+01	3.029E+02	2.166E+02	7.951E+02	8.354E+02	3.771E+00	8.546E+05	3.191E+01
TCM22 (18)	4.867E-01	6.548E+01	9.412E+00	B.D.L.	2.354E-01	9.065E-01	2.087E-01	2.402E+00	1.816E+00	6.574E+03	1.365E+04	9.310E+04	1.769E+03	2.108E+04	8.630E+03	1.533E+01	2.812E+00	2.741E+02	1.809E+03	4.169E+00	1.393E+01	2.108E+01	1.584E+02	6.587E+01	2.028E+00	1.454E+05	9.085E+00
TCM25 (19)	1.446E-01	7.512E+02	1.539E+01	2.159E-01	5.671E+00	1.840E+00	4.643E-01	2.257E+01	B.D.L.	6.564E+02	7.030E+03	8.821E+04	3.486E+03	8.066E+04	1.056E+05	8.333E+00	1.213E+01	1.592E+03	6.018E+03	7.499E+00	1.885E+01	2.065E+02	8.265E+02	5.219E+02	3.119E+00	2.642E+05	7.858E+00
TCM19 (20)	1.111E+00	5.648E+02	2.422E+01	1.038E+00	9.810E+00	3.922E+01	2.036E+00	1.815E+02	B.D.L.	5.295E+02	3.164E+04	6.675E+04	4.615E+04	5.326E+04	2.335E+05	4.770E+01	4.901E+01	8.718E+03	3.293E+04	8.647E+01	6.424E+01	2.730E+02	1.312E+03	6.541E+02	3.344E+00	6.351E+05	1.325E+04
TCM15 (21)	B.D.L.	1.144E+03	1.900E+01	3.835E-01	6.242E-01	1.903E+00	4.849E-01	6.421E+00	2.754E+00	5.752E+02	4.207E+04	4.350E+04	3.028E+03	3.426E+04	8.513E+04	4.995E+00	1.912E+01	3.971E+02	4.794E+03	4.272E+00	2.457E+01	1.533E+02	6.393E+02	2.102E+02	3.225E+00	1.982E+05	4.606E+01
TCM7 (22)	1.794E-01	8.321E+02	2.146E+01	1.084E-01	6.818E+00	1.726E+00	8.988E-01	2.986E+01	2.149E+01	9.579E+02	5.790E+04	7.445E+04	4.489E+03	1.037E+05	7.065E+04	1.790E+01	1.332E+01	1.950E+03	4.937E+03	1.824E+01	3.890E+01	2.006E+02	7.064E+02	5.193E+02	2.376E+00	4.451E+05	2.466E+01
TCM13 (23)	2.655E-01	1.137E+03	1.904E+01	1.153E-01	3.915E+00	1.408E+00	6.139E-01	4.110E+01	B.D.L.	6.843E+02	7.706E+03	5.571E+04	1.234E+04	6.417E+04	9.103E+04	1.265E+01	2.730E+01	3.098E+03	6.336E+03	1.021E+01	3.911E+01	1.961E+02	7.141E+02	6.101E+02	2.243E+00	2.636E+05	1.268E+01
TCM10 (24)	B.D.L.	7.180E+01	6.608E+00	B.D.L.	B.D.L.	7.895E-01	3.747E-02	1.143E+02	B.D.L.	3.177E+02	2.033E+03	8.783E+03	1.766E+02	1.038E+03	1.698E+04	3.043E-01	2.427E+00	8.928E+01	2.865E+02	8.441E-01	6.610E+01	8.167E+00	5.248E+01	1.323E+01	1.709E+00	3.759E+04	1.748E+00
TCM6 (25)	8.746E-02	9.312E+02	1.472E+01	2.851E-01	2.004E+00	1.675E+00	4.720E-01	1.115E+01	3.663E+00	8.700E+02	2.679E+04	6.300E+04	4.272E+03	1.034E+05	5.854E+04	1.388E+01	4.664E+01	7.000E+02	5.562E+03	7.360E+00	3.633E+02	2.499E+02	6.836E+02	9.267E+02	4.152E+00	3.859E+05	1.044E+01

B.D.L. Denotes that the concentration of an element in this sample is Below Detection Limits
